# The impact of college students’ parent–child attachment on bullying behavior: the mediating role of external expression of anger

**DOI:** 10.3389/fpsyg.2024.1467625

**Published:** 2024-12-11

**Authors:** Ruixin Wang, Yiwen Chen, Zhenyu Zhao, Mengmeng Zhao, Ziying Wang, Hongge Luo, Lina Li

**Affiliations:** School of Psychology and Mental Health, North China University of Science and Technology, Tangshan, Hebei, China

**Keywords:** parent–child attachment, external expression of anger, bullying behavior, college students, mediation effect

## Abstract

**Purpose:**

(1) To investigate the relationship between college students’ parent–child attachment, external expression of anger, and bullying behavior; (2) To explore the mediating role of external expression of anger between parent–child attachment and bullying behavior.

**Methods:**

The Parent–Child Attachment Scale, State–Trait Anger Expression Inventory, and Bullying Participation Behavior Questionnaire were administered to 306 college students. Data collected were analyzed using SPSS 26.0 for common method bias tests, descriptive analysis, and correlation analysis. The mediation model was tested using the PROCESS macro program.

**Results:**

Parent–child attachment was significantly negatively correlated with external expression of anger and bullying behavior, while external expression of anger was positively correlated with bullying behavior. Additionally, the external expression of anger partially mediated the impact of parent–child attachment on bullying behavior.

**Conclusion:**

This study clarifies the relationship between parent–child attachment, external expression of anger, and bullying, emphasizing the indirect effect of parent–child attachment on individual bullying behavior through the external expression of anger. It provides data support for the further development of innovative methods to effectively reduce bullying behavior.

## Introduction

Bullying behavior is the intentional and continuous infliction of harm by a perpetrator to a victim, which includes not only physical damage but also encompasses psychological negative impacts and adaptive issues (Olweus, 2003). Studies have shown that the incidence of school bullying in China has reached 19.1% (Chan and Wong, 2017; Zhang et al., 2019), among which the rate of college student bullying is as high as 8.03% (Yu et al., 2022). As a complex and multifaceted social issue, school bullying takes various forms, including physical and verbal bullying, and has a non-negligible impact on the psychological and physical well-being of college students who are in a critical period of social adaptation (Setiadi et al., 2021; Zhu, 2021). The repetitive and group nature of bullying exacerbates the long-term psychological trauma suffered by victims. Bullying experiences not only lead to a decline in academic performance and quality of daily life (Ren, 2017) but also diminish the individual’s sense of safety and well-being (Li et al., 2023). Individuals who are bullied over the long term may experience fear, anxiety, depression, and other psychological issues, potentially evolving into more severe mental health crises, leading to suicidal behavior (Sun and Liu, 2022; Ying et al., 2024). Moreover, school bullying also has a significant negative impact on the bullies themselves, affecting aspects such as personality, academics, behavior, and criminality (Liu et al., 2023). Therefore, it is crucial to delve into the influencing factors and mechanisms of bullying behavior to enhance understanding of school bullying, early identification of factors that may reduce the occurrence of bullying behavior, and timely intervention.

Parent–child attachment is an important protective factor in preventing bullying behavior. Bowlby (1982) first proposed in attachment theory that attachment refers to an emotional state that arises in the process of children’s communication and interaction with their parents. This state is continuously developing and not easily changed. As a biological mechanism formed in the process of evolution, attachment aims to protect children from harm and emphasizes the importance of the deep emotional bond established between family caregivers and children in the individual’s growth process (Ainsworth and Bowlby, 1991). The dual-harm cognitive-emotional regulation model suggests that individuals engage in bullying behavior to achieve emotional regulation or to maintain interpersonal functions (Shafti et al., 2021). Research indicates that for individuals with high attachment levels, stress can be regulated and external difficulties can be overcome, and they have stronger empathy and can apply more mitigation strategies in conflicts (Overall and Simpson, 2015). Therefore, individuals with higher levels of parent–child attachment are often able to deal with interpersonal problems or regulate emotions in a more positive way, rather than choosing to bully. The results of some studies also support this view, that the quality of parent–child attachment has a significant impact on whether adolescents are involved in bullying behavior, and a high level of parent–child attachment can reduce the likelihood of adolescents engaging in bullying behavior (Yu et al., 2023; Zhao, 2023; Zhu, 2021). It is evident that understanding the mechanism of college student bullying behavior from the perspective of parent–child attachment is of great significance. Therefore, we propose Hypothesis 1: Parent–child attachment is significantly negatively correlated with college student bullying behavior.

Additionally, the potential mediating role of external expression of anger between attachment and bullying behavior cannot be ignored. External expression of anger refers to the way individuals handle their anger, involving how they communicate their anger to the outside world, and pointing to potentially aggressive behaviors that include physical attacks or verbal insults, which are direct factors affecting the occurrence of bullying behavior (Spielberger et al., 1988). The self-control resource theory suggests that when individuals engage in self-control activities, they consume their internal self-control resources, and once these resources are depleted to a certain extent—leading to self-control failure—then negative emotions are more likely to be expressed outwardly (Tuk et al., 2015). The emotional support provided by a high level of parent–child attachment can promote the construction of positive psychological resources within the individual (Hobfoll, 2011). Therefore, individuals who feel secure in their relationships express less anger than those who feel insecure (He, 2021). In other words, parent–child attachment may be able to reduce the occurrence of bullying behavior by affecting the external expression of anger. Social learning theory emphasizes that children learn emotional expression by imitating their parents’ behavior. In high-level parent–child attachment, parents invest more in understanding rather than violence, and individuals feel more positive ways of expression, thus incline towards effectively solving problems and resolving emotions when they feel angry, rather than venting dissatisfaction through external bullying. (Brown and Dunn, 1996). Based on this, this study focuses on the potential mediating role of external expression of anger in the relationship between parent–child attachment and bullying, attempting to provide a new perspective for possible intervention measures to reduce bullying behavior. And we propose Hypothesis 2: External expression of anger mediates the relationship between parent–child attachment and bullying behavior.

It is worth noting that some studies have indicated that parental divorce may affect an individual’s level of bullying behavior (Li, 2023), and this needs to be considered as a control variable. Specifically, when factors such as parental divorce, poor relationship quality, or an unhealthy family atmosphere are present, students may experience psychological insecurity and loneliness, often finding themselves in a vulnerable position in interpersonal relationships. To protect themselves, they may act more aggressively when conflicts arise with others, leading to aggressive bullying behavior (Huang et al., 2024). Additionally, some research has pointed out that gender differences can affect the level of external expression of anger (Timmers et al., 1998), and whether being an only child can affect the level of parent–child attachment (Chen, 2023). Therefore, we will also consider gender and whether being an only child as control variables.

In summary, this study attempts to explore the relationship between parent–child attachment and bullying behavior, as well as the mediating role of external expression of anger in the relationship between parent–child attachment and bullying behavior, in order to provide data support for targeted educational and intervention measures. And we propose two hypotheses.

*H1*: There is a significant negative correlation between college students’ parent–child attachment and school bullying behavior.

*H2*: The external expression of anger among college students serves as a mediator between parent–child attachment and school bullying behavior.

## Methods

### Participants

Between June and September 2024, this study conducted a survey among 320 college students from a certain university using convenient sampling. Among the collected questionnaires, 14 were identified as invalid due to missing answers or patterned responses and were therefore excluded. A total of 306 valid questionnaires were obtained, with a valid recovery rate of 95.63%. Among them, there were 89 male college students (29.08%) and 217 female college students (70.92%); 90 students were only children (29.41%), and 216 students were not only children (70.59%); 29 students had divorced parents (9.48%), and 277 students had non-divorced parents (90.52%) (Table 1).

**Table 1:** Sample characteristics.

Sample characteristic	Undergraduated participants
*N*	306
Sex	
Males	89 (29.08%)
Females	217 (70.92%)
The number of children in the family	
One-child	90 (29.41%)
Multiple children	216 (70.59%)
Parental marital status	
In marriage	227 (90.52%)
Divorced	29 (9.48%)

### Measures

#### Parent–child attachment

In this study, the Parent and Peer Attachment Questionnaire developed by Armsden and Greenberg (1987) was used to measure parent–child attachment, specifically the parent attachment section of the scale. Two subscales were selected: father attachment and mother attachment. Each subscale is divided into three dimensions: communication, trust, and alienation, with 25 items in each dimension. Sample items include “My mother respects my feelings.” The responses are rated on a 5-point scale, with 1 point indicating “never” and 5 points indicating “always.” Higher scores indicate better quality of parent–child attachment. In this study, the father attachment subscale had a Cronbach’s *α* of 0.94, the mother attachment subscale had a Cronbach’s α of 0.92, and the total scale had a Cronbach’s α coefficient of 0.95, indicating good internal consistency.

#### External expression of anger

The State–Trait Anger Expression Inventory-2 (STAXI-2), developed by Spielberger et al. (1988) and colleagues, consists of three subscales designed to assess an individual’s anger experience, expression, and control, with a total of 57 items. The Chinese version of the scale was revised by Tao (2009). This study utilized the Anger Expression and Control Scale, which includes items such as “When I am angry or furious, my usual reaction is: I show it.” The ratings are made on a 4-point scale, with 1 point indicating “almost never” and 4 points indicating “almost always.” The level of external expression of anger for an individual is determined by the total score of all items, and the Cronbach’s *α* coefficient for this study is 0.70.

#### Bullying behavior

The Bullying Participation Behavior Questionnaire (BPBQ) — Bullying Behavior Subscale: Revised by Qiu et al. (2020) and colleagues, this scale consists of 10 items rated on a Likert scale of 5 points, ranging from the lowest score of 1 (“Never”) to the highest score of 5 (“7 times or more”). An individual’s level of bullying behavior is determined by the total score of all items, with higher scores on the questionnaire indicating a higher frequency of involvement in bullying behavior. The Cronbach’s α coefficient for this study is 0.82.

### Data analysis

In this study, SPSS 26.0 was used to conduct a common method bias test on the collected data. The results of Harman’s single-factor test indicated no significant bias (the ratio of the first factor was 28.50%, which is less than 40%). Furthermore, the study utilized SPSS 26.0 to perform descriptive statistics and Pearson correlation analysis on the collected data. The mediation model was tested using Model 4 of the PROCESS macro program, with the criterion for significance set at *p* < 0.05.

## Results

### Preliminary analysis

The results of the Pearson correlation analysis are as follows (see Table 2), Parent–child attachment is significantly correlated with bullying behavior and external expression of anger (*r* = −0.28, −0.27, *p* < 0.01). Bullying behavior is significantly positively correlated with external expression of anger (*r* = 0.43, *p* < 0.01). Parent–child attachment shows a correlation with being an only child (*r* = −0.13, *p* < 0.1). Bullying behavior is correlated with whether the parents are divorced (*r* = −0.23, *p* < 0.01). External expression of anger is correlated with gender, whether being an only child, and whether the parents are divorced (*r* = 0.12, *p* < 0.1; *r* = 0.04, *p* < 0.1; *r* = 0.12, *p* < 0.01).

**Table 2:** Correlation analysis (*r*).

	1	2	3	4	5	6
1. Sex	1					
2. The number of children in the family	–	1				
3. Parental marital status in marriage divorced	–	–	1			
4. Parent–child attachment	−0.05	−0.13*	0.22**	1		
5. Bullying behavior	−0.02	−0.04	−0.23**	−0.28**	1	
6. External expression of anger	0.12*	0.04*	−0.19**	−0.27**	0.43**	1
*M*				172.66	15.42	15.88
*SD*				30.05	3.43	4.85

*p < 0.05, **p < 0.01.

### The mediating effect of bullying behavior

After the correlation analysis revealed significant correlations between parent–child attachment, bullying behavior, external expression of anger, and demographic variables such as gender, whether being an only child, and parental divorce status, dummy variable treatment was applied to control for these factors before testing the mediating effect. These variables were standardized and then analyzed using Model 4 of the PROCESS macro program in SPSS software to examine the mediating role of external expression of anger between parent–child attachment and bullying behavior (see Table 3). The results showed (see Table 3) that parent–child attachment had a significant negative predictive effect on both external expression of anger and bullying behavior (*β* = −0.24, −0.17, *p* < 0.001), indicating that higher quality of parent–child attachment is associated with lower tendencies of external expression of anger and bullying behavior. Additionally, the external expression of anger had a significant positive predictive effect on bullying behavior (*β* = 0.38, *p* < 0.001).

**Table 3:** The examination of the mediating effect of the external expression of anger between parent–child attachment and bullying behavior.

Outcome variables	Predictors	*R*	*R^2^*	*F*	*β*	*t*
External expression of anger		0.31	0.10	8.27***		
	Sex 1				−0.22	−1.84
	One-child 1				−0.03	−0.24
	Divorced 1				0.45	2.31*
	Parent–child attachment				−0.24	−4.09***
Bullying behavior		0.49	0.24	0.80***		
	Sex 1				0.16	1.38
	One-child 1				0.12	1.09
	Divorced 1				0.43	2.36
	Parent–child attachment				−0.17	−3.14**
	External expression of Anger				0.38	7.04***
Bullying behavior		0.34	0.12	9.77***		
	Sex 1				0.07	0.59
	One-child 1				0.11	0.92
	Divorced 1				0.60	3.08**
	Parent–child attachment				−0.26	−4.53***

*p < 0.05, **p < 0.01, ***p < 0.001.

**Figure 1:**
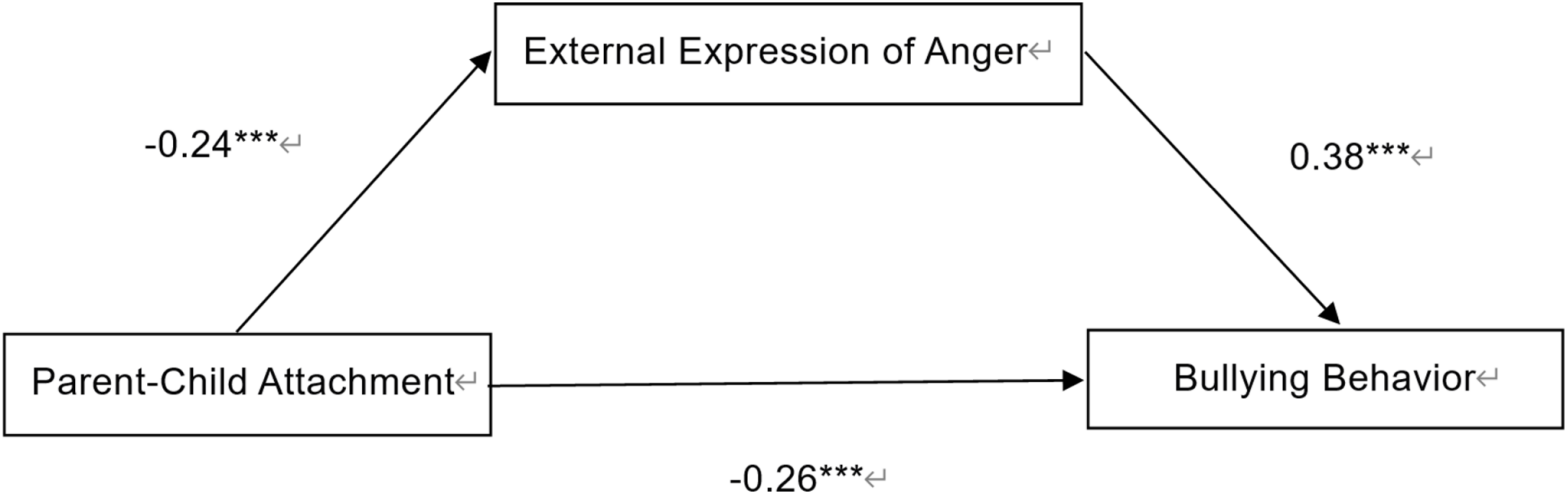
Mediating pathways of external expression of anger.

When external expression of anger was included as a mediator in the model, the effect of parent–child attachment on bullying behavior remained significant (*β* = −0.17, *p* < 0.01). Therefore, the external expression of anger played a partial mediating role between parent–child attachment and bullying behavior, with a mediation effect value of 0.33, and its 95% confidence interval was [−0.14, −0.05], indicating that this mediating effect is significant and accounts for 33.33% of the total effect (see Table 4). The mediation model was established, as shown in Figure 1.

**Table 4:** The decomposition diagram of total effect, direct effect, and mediating effect.

	Effect size	*BootSE*	Boot CI	Relative effect size
Total effect	−0.27	0.06	[−0.38,-0.15]	
Direct effects	−0.18	0.06	[−0.28,-0.07]	66.7%
Indirect effects	−0.09	0.02	[−0.14,-0.05]	33.3%

## Discussion

### The correlation between parent–child attachment, external expression of anger, and bullying behavior

This study demonstrates that parent–child attachment and external expression of anger are closely related to bullying behavior among college students. The higher the level of parent–child attachment, the lower the level of external expression of anger, and consequently, the lower the level of bullying behavior. Additionally, the external expression of anger plays a partial mediating role between parent–child attachment and college student bullying behavior. Parent–child attachment not only has a direct effect on bullying behavior but also indirectly affects bullying through the external expression of anger. The results of this study validate attachment theory and support the consideration of parent–child attachment as a protective factor against bullying behavior, providing a new perspective and approach for predicting and controlling the occurrence of bullying behavior.

The findings regarding the link between parent–child attachment and bullying behavior are consistent with previous studies (Xu, 2019), Hypothesis 1 has been validated, indicating that the closer the parent–child attachment, the less bullying behavior individuals exhibit. According to attachment theory, adolescents with a secure attachment style are more likely to trust others, maintain an optimistic attitude when facing challenges, and adopt positive values and behaviors (Mikulincer et al., 1998). This positive attitude contributes to the individual’s overall development, including psychological health. In contrast, a low level of attachment may lead to distrust and indifference in adolescents, affecting their social skills and increasing the risk of violent and avoidant behaviors (Huang and Song, 2017). When studying the causes of youth bullying behavior, the parent–child relationship is found to play a crucial role, with a healthy parent–child attachment relationship helping to reduce the likelihood of a child’s involvement in school bullying; conversely, if the parent–child attachment is impaired, children are more likely to frequently engage in bullying behavior (Zhu, 2021). This finding emphasizes the importance of establishing and maintaining healthy parent–child relationships to prevent and reduce the occurrence of school bullying incidents.

Furthermore, the study shows that the level of parent–child attachment is negatively correlated with the level of external expression of anger, which is consistent with previous research findings (Mo, 2019). Specifically, individuals with low attachment levels are more likely to externalize their anger. Moreover, there is a positive correlation between the external expression of anger and bullying behavior. Bowlby (1982) pointed out that an individual’s attachment anxiety not only affects their negative self-evaluation but also their positive view of others, which may lead individuals to defend themselves through the expression of anger when they feel abandoned or suspicious. The positive correlation between the external expression of anger and bullying behavior may be driven by various factors. Bullying behavior often involves the abuse of power and intentional harm to others, and the external expression of anger may make individuals more inclined to adopt aggressive behaviors, thus becoming bullies (Kochenderfer-Ladd and Wardrop, 2010).

It is noteworthy that the analysis results reveal the relationship between gender, whether being an only child, and parental divorce with the three research variables. Specifically, students from divorced families scored higher on bullying behavior. This is consistent with existing research (Li, 2024; Liu, 2024; Zeng et al., 2024). Children from single-parent families, due to facing more adverse growth environments, usually have lower quality parent–child attachment than children from intact families (He, 2021). In the case of divorce, children usually live with only one parent, which may lead to the absence and tension in parent–child relationships, thereby affecting children’s psychological health and behavioral development (Zhang, 2023; Zhang and Wu, 2024), making them more likely to exhibit bullying behavior (Yu et al., 2023). Additionally, gender is significantly related to the external expression of anger, with female college students expressing anger externally at a significantly higher level than males. This difference may stem from different social and cultural rules for emotional expression between genders: females are allowed to express more, stronger, and more exaggerated emotions (Li, 2024), while males are more inclined to suppress emotional expression (Wang et al., 2024). Under this cultural background, the differences in emotional expression between men and women are formed. However, some studies have not found significant gender differences in anger expression (Hou et al., 2017), indicating that conclusions on this issue are not yet unified and require further exploration in future research. Lastly, being an only child is related to the level of parent–child attachment among college students, with only children having significantly higher levels of parent–child attachment than non-only children, a result consistent with previous research (Chen, 2023). Only children enjoy all the love and attention of their parents, have more opportunities for effective communication and interaction with their parents, and are more likely to establish a stronger trust relationship. In contrast, in families with multiple children, parents’ attention and resources need to be distributed among several children, which may lead to a reduction in the intimacy and understanding between parents and children (Liu, 2024).

The results of the correlation analysis suggest the importance of focusing on creating a good family environment, establishing high-quality parent–child attachment, and guiding individuals to understand the correct ways of expressing anger to reduce the potential significance of bullying behavior. It requires families and schools to prevent bullying behavior from the perspective of parent–child attachment and anger expression methods, while also indicating the need for targeted interventions for individuals of different genders and family situations.

### The mediating role of the external expression of anger

This study found that the external expression of anger partially mediates the impact of parent–child attachment on bullying behavior. Parent–child attachment not only directly predicts bullying behavior but also indirectly affects it through the external expression of anger, as for Hypothesis 2. College students with low attachment levels are more likely to transform negative emotions towards others into anger and express them externally through bullying behavior. Attachment theory suggests that the level of attachment can influence an individual’s way of expressing anger. The expression of anger is not just for venting revenge or even destroying relationships with others; it is more a tool to convey one’s dissatisfaction and unfair treatment to the other party (He, 2021). Individuals with low attachment levels may tend to express anger externally in the hope of achieving a better attachment relationship (Mikulincer and Shaver, 2011). Moreover, a good family environment is an essential background for cultivating individuals’ appropriate emotional regulation abilities. High levels of parent–child attachment bring more positive characteristics that help children develop effective emotional regulation skills. In healthy parent–child relationships, parents are usually more sensitive and responsive to their children’s emotional reactions. This environment promotes the development of children’s emotional regulation abilities, enabling them to handle anger in more adaptive ways. In contrast, low levels of parent–child attachment may lead to difficulties in emotional regulation, influencing the expression of anger (Ainsworth et al., 1978; Yu et al., 2023), making individuals with low attachment levels more likely to choose direct conflict and violence to express anger, such as bullying (Mikulincer and Shaver, 2007; Spielberger, 1995). In problematic parent–child relationships (e.g., parental divorce), children may be more inclined to handle interpersonal relationships violently due to insecurity or to protect their status in interpersonal relationships, which is associated with an increased risk of bullying behavior (Pan et al., 2024).

Starting from the perspective of anger expression style, this study explores the mediating role of the variable external expression of anger between parent–child attachment and bullying behavior, further expanding and deepening previous research, and contributing to the understanding of how parent–child attachment “affects” bullying behavior, enriching the study of bullying behavior. The results highlight the importance of enhancing parent–child communication, encouraging parents to pay attention to emotional management education, establishing open communication with their children, regularly engaging in emotional exchanges, creating a warm and safe family environment, strengthening children’s sense of attachment security, helping children express and manage anger emotions, and avoiding the venting of emotions through bullying behavior. For individuals who have not established a good parent–child attachment in the family, schools and society can start with anger expression styles, strengthen emotional management and mental health education, help students learn to effectively deal with anger emotions, and promptly identify and intervene in bullying behavior.

### Limits and contributions

This study explored the relationships among college students’ parent–child attachment, external expression of anger, and bullying behavior, as well as the underlying mechanisms, aiming to provide theoretical guidance and empirical evidence for the prevention and intervention of bullying behavior among college students. However, there are still deficiencies. Firstly, this study entirely relied on the self-reports of college students, and individuals’ perceptions of themselves can be biased. Future studies could collect data from multiple aspects, such as through observations or reports from peers, to enhance the accuracy and reliability of the data.

Secondly, this cross-sectional study does not allow for the testing of causal relationships between college students’ bullying behavior and the external expression of anger. It is currently unclear whether students’ bullying behavior could, in turn, affect the external expression of anger. Therefore, future longitudinal studies are needed to examine causality.

Thirdly, the convenience sampling method used in this study limits the representativeness of the sample. This limitation emphasizes the need for caution when generalizing the results. To improve the comprehensiveness and reliability of the research findings, future studies should consider expanding the sampling range and increasing the sample size.

Fourth, the mediation model constructed in this study uses the external expression of anger as a mediating variable to explore the connection between parent–child attachment and school bullying. However, whether there are other potential variables affecting this relationship still requires further investigation. Future research could include more variables to more comprehensively explore the relationship between parent–child attachment and school bullying.

## Conclusion

The study indicates that the higher the level of parent–child attachment among college students, the lower the degree of external expression of anger, and consequently, the lower the likelihood of engaging in bullying behavior. Parent–child attachment not only directly predicts school bullying but also has an indirect impact on bullying behavior through the external expression of anger. While this finding may not directly improve the current situation, it can help us better understand the mechanisms behind this phenomenon and explain the influence of family education on individual psychological health and school life.

## Data Availability

The original contributions presented in the study are included in the article/supplementary material, further inquiries can be directed to the corresponding authors.
